# Transmission and clinical characteristics of asymptomatic patients with SARS-CoV-2 infection

**DOI:** 10.2217/fvl-2020-0087

**Published:** 2020-06-12

**Authors:** Jie Tan, Shousheng Liu, Likun Zhuang, Lizhen Chen, Mengzhen Dong, Jie Zhang, Yongning Xin

**Affiliations:** 1 ^1^School of Clinical Medicine, Weifang Medical University, Weifang, 261053, China; 2 ^2^Department of Infectious Disease, Qingdao Municipal Hospital, Qingdao, 266011, China; 3 ^3^Central Laboratories, Qingdao Municipal Hospital, Qingdao, 266011, China; 4 ^4^Hepatology Laboratory, Qingdao Municipal Hospital, Qingdao, 266011, China

**Keywords:** asymptomatic infections, COVID-19, epidemic, SARS-CoV-2

## Abstract

The 2019 novel coronavirus disease, SARS-CoV-2, is now spreading globally and is characterized by person-to-person transmission. However, it has recently been found that individuals infected with SARS-CoV-2 can be asymptomatic, and simultaneously a source of infection in others. The viral load detected in nasopharyngeal swabs of asymptomatic carriers is relatively high, with a great potential for transmission. More attention should be paid to the insidious spread of disease and harm contributed by asymptomatic SARS-CoV-2 carriers. To provide a theoretical basis for the accurate and early clinical identification of asymptomatic patients, this review objectively summarizes the epidemic status, transmission characteristics and clinical features of asymptomatic patients with SARS-CoV-2 infection.

## Background

COVID-19 caused by SARS-CoV-2, formerly 2019 novel coronavirus or 2019-nCoV [1], broke out in Wuhan, China in December 2019 [2]. Epidemics of COVID-19 are now occurring worldwide [3]. Since the first COVID-19 case in Wuhan was identified on 12 December 2019, in less than 4 months (1 April 2020), the number of cumulative confirmed cases in the world has exceeded 800,000 [4,5]. On 30 January 2020, the outbreak was declared an international public health emergency by the World Health Organization (WHO) [6].

SARS-CoV-2 is not the first coronavirus to threaten the life and welfare of humans. The years 2002 and 2012 saw the emergence of SARS-CoV and MERS-CoV [7]. Like SARS-CoV-2, the transmission of SARS-CoV or MERS-CoV is person-to-person [8–11], but they differ in crucial aspects. Most patients infected with SARS-CoV present with obvious clinical symptoms within a short period, the disease progresses rapidly and peak viral shedding occurs in the late stage [8]; few patients with SARS are asymptomatic [11]. MERS is primarily a zoonotic disease, and spread among humans was scattered and limited, the symptoms were obvious and infection rarely preceded symptom onset. Nosocomial transmission was more troublesome than community spread [8,12].

In contrast, onset of SARS-CoV-2 is insidious. When asymptomatic or the early symptoms are mild, patients can move freely and transmit the virus [13], with an incubation period that is long and infectious [14,15]. These characteristics allow for easy spread, and infection sources can be difficult to identify and isolate. In addition, the main routes of transmission are through respiratory droplets and contact [16], which is relatively easy to achieve [17].

At present, almost all countries in the world have recognized the seriousness of the COVID-19 pandemic and implemented various measures to curb its development, but asymptomatic patients are not always taken seriously by healthcare workers. Yet, asymptomatic infections of SARS-CoV-2 are probably an important source of transmission. The person with an asymptomatic confirmed case of infection has normal body temperature or is only slightly indisposed [18].

These differences between COVID-19 and SARS or MERS require a change in epidemic response plan. Only by fully researching the various characteristics and mechanisms of asymptomatic infections can we lay a theoretical foundation for deployment of the next steps in its control. With that purpose, this review summarizes the epidemic status, transmission characteristics and clinical features of asymptomatic patients with SARS-CoV-2 infection.

## Epidemic status & transmission characteristics of asymptomatic COVID-19 patients

### Confirmed asymptomatic SARS-CoV-2 infections continue to increase

**Figure 1.**
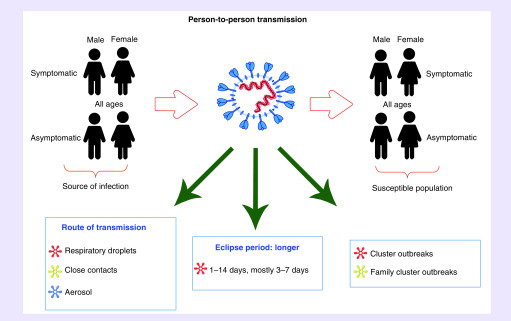
Transmission characteristics of SARS-CoV-2. The SARS-CoV-2 possesses the characteristics of person-to-person transmission. The source of infection and susceptible population exist in both the male and female of any age, and the disease performance is both symptomatic and asymptomatic. The SARS-CoV-2 is mainly transmitted through respiratory droplets and close contact; furthermore, there is a possibility of aerosol transmission when it is in a relatively closed environment and exposed to high concentrations of aerosol for a long time. The SARS-CoV-2 has an incubation period of 1–14 days, but mostly range 3–7 days. In addition, many studies have confirmed the existence of a large number of cluster outbreaks and family cluster outbreaks.

Multiple studies indicate that asymptomatic infections make up a large percentage of confirmed COVID-19 cases. A retrospective study in Beijing collected data for 262 individuals with diagnosed COVID-19 from 20 January to 10 February 2020, and 13 were asymptomatic (5.0%) [18]. In addition, 126 persons of German nationality were evacuated from Hubei Province to Frankfurt, Germany, on 1 February 2020. After strict screening and testing, two were confirmed to have SARS-CoV-2 infection, yet both patients were asymptomatic [19]. A further epidemiological investigation (28 January to 9 February 2020) was conducted in clinics and communities in Nanjing, Jiangsu Province, China. The survey screened the close contacts of patients with confirmed or suspected infections. The results of nucleic acid screening identified 24 confirmed SARS-CoV-2 carriers without any obvious symptoms. Of these, five patients developed typical symptoms during the subsequent hospitalization, while the other 19 patients remained asymptomatic [15]. Furthermore, the Ministry of Health, Labor and Welfare of Japan announced on 5 March 2020 that among 696 people on the ‘Diamond Princess’ cruise ship infected with SARS-CoV-2, 410 were asymptomatic [20]. All of the above indicates that in the community there may be a large number of unidentified asymptomatic people with contagious infections (Figure 1).

### Asymptomatic patients with SARS-CoV-2 infection may carry high viral loads

SARS-CoV-2 has been detected in nasopharyngeal swabs and sputum samples from asymptomatic patients [11]. The viral load detected in asymptomatic individuals was similar to that of symptomatic patients suggesting that people without symptoms have a strong ability to transmit the virus to others [21]. In addition, SARS-CoV-2 has been detected in the blood and stool samples of seemingly well patients [22–24], and compared with the virus in respiratory secretions, the virus in feces may take longer to clear [25].

### Complex incubation period in asymptomatic SARS-CoV-2 infection

In general, patients with symptoms of SARS-CoV-2 infection are admitted to hospital for detection and treatment under isolation. However, asymptomatic individuals may not be recognized by healthcare workers, and do not self-isolate or seek treatment. Bai *et al.* [14] showed that the incubation period of an asymptomatic patient was 19 days. What is more, Hu *et al.* [15] reported that the communicable period of asymptomatic COVID-19 patient may be as high as 29 days.

### Transmission of SARS-CoV-2 by asymptomatic persons is implicated in crowd & family-clustered outbreaks

Multiple studies have found that there are asymptomatic SARS-CoV-2 infections in the process of crowds and family-clustered outbreaks. Among a family of six in Shenzhen who traveled to Wuhan from 29 December 2019 to 4 January 2020, five members were identified with COVID-19, including an asymptomatic 10-year-old boy [11]. A family of three who traveled on 22 January 2020 from Wuhan to Guangzhou, China, through the high-speed rail tested positive for SARS-CoV-2, but only one developed clinical symptoms, and the other two members had no signs or clinical symptoms [26]. Infants also are not spared from SARS-CoV-2 infection. The first pediatric case was confirmed asymptomatic in Singapore. The infant was part of a family transmission cluster, in which its parents and their live-in helper were symptomatic [22]. Furthermore, asymptomatic COVID-19 patients can even become the source of infection in contagious outbreaks among families. SARS-CoV-2 transmission from an asymptomatic infected person returning home from Wuhan on 10 January 2020 was suspected as the cause of a family cluster epidemic of five members in Anyang, China [14]. In fact, any infected person, symptomatic or asymptomatic, may be the first to transmit SARS-CoV-2 to other members in a clustered and family-clustered outbreak.

## Clinical characteristics of asymptomatic patients with SARS-CoV-2 infection

### Identification & diagnosis of asymptomatic SARS-CoV-2 infection

At present, cases of COVID-19 continue to occur around the world, so the rate of asymptomatic infections cannot be accurately determined. Identification and isolation of asymptomatic patients is essential to control virus outbreaks. Various studies have shown that asymptomatic persons with SARS-CoV-2 infection are generally not discovered until after their families, relatives, friends or close contacts have symptoms that are diagnosed [11,14,21,22,26–28]. Therefore, in order to not miss any infected patients, it is best to perform screening for all close contacts of patients with confirmed or suspected infections [15,19]. The main tests used to diagnosis COVID-19 are the SARS-CoV-2 nucleic acid test (NAT) of nasopharyngeal swab samples, the SARS-CoV-2 specific serological test and chest computed tomography (CT) scanning. NAT by reverse transcription-PCR (RT-PCR) is well established as the gold standard for the diagnosis of COVID-19 [29], but still the test is associated with false negatives due to problems with sample collection and the operating procedures [30,31]. Therefore, for cases that are highly suspicious of COVID-19 but test negatively by NAT, diagnosis via screening with a SARS-CoV-2 specific serological test and chest CT scan may be of great value [28,32,33]. In one study of 285 COVID-19 patients with acute antibody responses to SARS-CoV-2, in 19 days after the onset of symptoms, 100% of the patients were positive for antiviral IgG. Importantly, the seroconversion of IgG and IgM occurs simultaneously or sequentially, and the titers of IgG and IgM were found to be stable within 6 days after seroconversion [34]. However, if the NAT result is negative and the SARS-CoV-2 specific serological test is positive, the diagnosis still cannot be directly confirmed. It is necessary to continue to observe and conduct multiple NAT tests until either the NAT result is positive or the SARS-CoV-2 specific serological test is determined to be a false positive. A study conducted in the USA used 1020 serum specimens that were previously tested for HSV serology by western blotting in 2018 and 2019 (prior to SARS-CoV-2 circulation) and detected one false positive using the Abbott SARS-CoV-2 IgG test [35]. In addition, many studies on SARS-CoV-2 specific serological tests have shown that it is difficult to achieve 100% sensitivity and specificity [36,37]. Thus, for now, the diagnosis of COVID-19 remains a challenge globally. No test method is completely mature and reliable, but the combination of multiple testing methods can improve the effectiveness of screening [38] and avoid missed diagnoses and misdiagnoses as much as possible [33].

It is important to note that some patients with COVID-19 may experience only mild symptoms and signs. Kam *et al.* [22] reported a 6-month-old infant who developed a temperature of 38.5°C during hospitalization, although for only 1 h. Hoehl *et al.* [19] reported a 48-year-old German woman who experienced a mild rash and minimal pharyngitis after admission. Therefore, persons with COVID-19 may appear essentially asymptomatic, but do experience very mild symptoms and can enter the recovery period without being detected. Therefore, at the time of consultation healthcare workers should thoroughly interview the patient for any recollection of discomfort.

### Variety of people with SARS-CoV-2 infection may be asymptomatic

Asymptomatic infection is not limited to young or middle-aged adults [21], but also children [11], infants [22] and even the elderly [27]. Hu *et al.* [15] showed that asymptomatic patients were relatively young, with a median age of 14 years in seven cases. In addition, asymptomatic infections were found in both males [19] and females [14].

### Disease progression, changes in CT images & laboratory indicators in people with asymptomatic SARS-CoV-2 infection

In general, asymptomatic infected people do not suffer seriously, but the virus they transmit can cause others to develop severe disease [15]. Those with asymptomatic infections did not always show lung changes such as ground glass opacities after CT examination, and may appear normal [28]. Yet, in other cases the typical changes in CT examination may be observed [27]. Changes in laboratory test indicators typical of SARS-CoV-2 infection have been found in some asymptomatic patients [11,22,27], but for others, indicators are normal (Table 1) [14,19,26].

**Table 1.** Clinical characteristics of asymptomatic patients with SARS-CoV-2 infection in recent studies.

Study	Country (patient)	Age (years)/sex	Chronic medical illness	Clinical characteristics			Collection site and viral load	Ref.
				Laboratory analysis	Computed tomography	Other situations		
Chan	China	10/Male	None	Alkaline phosphatase (↑)^†^	GGOs	NM	Nasopharyngeal swab (NF), throat swab (40)^‡^, sputum (27)^‡^	[11]
Bai	China	20/Female	NA	NOA	NOA	NOA	Nasopharyngeal swab (+)^§^	[14]
Hu	China	32.0^¶^ (15.0–57.0)	DMs (2)^#^	Blood leukocyte count (↓ 2)^#^^,^^††^	Normal (7)^#^	NOA	Pharyngeal swab (+)^§^	[15]
			Hypertension (2)^#^	Lymphocyte count (↓ 2)^#^^,^^††^	GGO or patchy shadowing (12)^#^			
		Male (8) Female (11)	CHD (1)^#^	C-reactive protein (↑ 2)^†^^,^^#^				
				Procalcitonin (↑ 4)^†^^,^^#^				
			CVD (1)^#^	Lactose dehydrogenase (↑ 3)^†^^,^^#^	stripe shadowing (5)^#^			
				Alanine aminotransferase (↑ 2)^†^^,^^#^				
				Creatinine (↑ 2)^†^^,^^#^				
				D-dimer (↑ 3)^†^^,^^#^				
Hoehl	Germany	58/Male	NA	Anemia	NM	NOA	Throat swab (24.39 and 30.25)^‡^	[19]
	Germany	48/Female	NA	NOA		Faint rash; Minimal pharyngitis		
Zou	China	26/Male	NA	NM	NOA	NOA	Nasal swab (22–28)^‡^ Throat swab (30–32)^‡^	[21]
Kam	Singapore	6-Month-old/male	NA	Neutropenia (day 8 of admission)	NP	Temperature rise (38.5°C in 1 h)	Nasopharyngeal swab (N gene 15.57; Orf1ab gene 13.73)^‡^ Blood sample and stool sample (+)^§^	[22]
Pan	China	33/Female	NA	NOA	NOA	NM	Nasopharyngeal swab: (+)^§^	[26]
		3/Male						
Lin	China	61/Male	None	C-reactive protein (↓)^††^	Multiple GGOs (day 1 of admission)	Only mild shortness of breath (1 day)	Throat swab (+)^§^	[27]
Bai SL	China	61/Male	CHD	NM	GGOs; lesion occupying lung field (different degree)	NOA	Throat swab (+)^§^	[28]
		53/Male	None					
		65/Female	DMs					
		34/Male	None					
		31/Female	None					

^†^ The indicator increased.

^‡^ CT value obtained by RT-PCR viral nucleic acid test.

^§^ Positive by RT-PCR viral nucleic acid test but CT value was not shown.

^¶^ Age, median-IQR.

^#^ Number of cases with an indicator or performance.

^††^ The indicator reduced.

CHD: Coronary heart disease; CoV: Coronavirus; CT: Computed tomography; Ct: Cycle threshold; CVD: Cerebrovascular disease; DM: Diabetes mellitus; GGO: Ground glass opacity; IQR: Interquartile range; NA: Not available; NF: No SARS-CoV-2 found; NM: Not mentioned; NOA: No obvious abnormality; NP: Not performed; RT: Reverse transcription.

## Conclusion

The number of people with COVID-19 continues to increase. Asymptomatic infections are hidden and easily overlooked. However, their potential to spread the virus cannot be underestimated, as the viral load they carried and their ability to infect close contacts may be similar to those of symptomatic individuals. In addition, asymptomatic infections can occur in any age range and either gender, and there may be no abnormalities in laboratory tests or CT examination. This, complete isolation of all sources of infection in the COVID-19 outbreak is a major problem. For this reason, measures of home quarantine and centralized isolation for observation over time have been and will continue to be necessary; otherwise, the pandemic will continue to cause great harm to the public and disease control will become even more complicated. Anyone who has had close contact with a confirmed or suspected case of COVID-19 should be closely monitored and screened; and therefore, the centralized isolation for medical observation and related tests of COVID-19 should be applied to the greatest extent possible, even if they have no symptoms. Healthcare workers should give close attention to screening consultations and collect detailed information, including the presence of even very slight symptoms. Overall, we have objectively summarized the current transmission and clinical characteristics of asymptomatic patients with COVID-19, which are deserve for further study and exploration in the future.

## Future perspective

At present, the number of confirmed cases of COVID-19 continues to increase, and various prevention and control measures continue to be needed. With more in-depth research on COVID-19, systematic treatment plans and guidelines have been improved, so it is particularly important for patients to be diagnosed early and admitted to hospital for isolation treatment. Unfortunately, asymptomatic patients with SARS-CoV-2 infection are a silent source of infection, who can unknowingly place others at risk for infection. Therefore, as more research is conducted to understand the mechanisms of SARS-CoV-2 infection and to develop treatment methods, efforts to prevent the transmission of SARS-CoV-2 by asymptomatic individuals will be the key to reducing the spread of COVID-19. While the epidemics of SARS, MERS and COVID-19, were all caused by coronaviruses and shared other similarities, there are many differences among these diseases as well. The number of cases of COVID-19 far exceeds the case numbers for the other epidemics, and of these three diseases, COVID-19 is the only one to cause a global pandemic. Notably, this, once insignificant and benign family of viruses, the coronavirus, has now generated three serious epidemics in the last two decades, indicating the importance of remaining alert to this class of emerging infectious diseases. This will be a long-term challenge, and we cannot yet predict when the next coronavirus outbreak will occur. What we can do is carry out more research and testing and develop better plans to handle such outbreaks in order to be better prepared when they emerge.

**Executive summary**

- The COVID-19 that originated in Wuhan, China in December 2019, is caused by SARS-CoV-2 infection. COVID-19 is now a global pandemic, and almost all countries in the world have recognized its seriousness and implemented various measures to curb its spread.
- The numbers of confirmed asymptomatic SARS-CoV-2 infections continue to increase, indicating that a large number of unidentified asymptomatic individuals with contagious infections may remain undetected in communities. In general, these patients do not know to self-isolate or seek treatment, and thus, are unlikely to be detected by healthcare workers. However, their potential to spread the virus cannot be underestimated, and emerging evidence indicates that they are an important source of transmission.
- SARS-CoV-2 has been detected in nasopharyngeal swabs, sputum samples, blood samples and stool samples from asymptomatic patients, and asymptomatic patients with SARS-CoV-2 infection can carry high viral loads.
- The incubation period of asymptomatic SARS-CoV-2 infections is complex and exists in the process of crowd and family-clustered outbreaks. In addition, asymptomatic patients are even the source of infection in contagious outbreaks among families.
- Asymptomatic infections can occur in patients of any age and either gender, and they may not exhibit any abnormalities on laboratory or computed tomography examinations.
- The identification and isolation of asymptomatic patients are essential to controlling virus outbreaks. To avoid missing any infected patients, anyone who has had close contact with a confirmed or suspected case of infection should be closely monitored and screened. Therefore, centralized isolation for medical observation and related tests for COVID-19 need to be applied to the greatest extent possible, even among contacts who have no symptoms. Healthcare workers should pay close attention to screening consultations and collect detailed information, including the presence of even very mild symptoms.
- Coronaviruses were once considered an insignificant, benign family of viral pathogens, but have now caused three major outbreaks of serious illness in the last two decades. Thus, we must all stay alert to such emerging infectious viral diseases, and this will be a long-term challenge.
